# A Flexible Reporter System for Direct Observation and Isolation of Cancer Stem Cells

**DOI:** 10.1016/j.stemcr.2014.11.002

**Published:** 2014-12-11

**Authors:** Binwu Tang, Asaf Raviv, Dominic Esposito, Kathleen C. Flanders, Catherine Daniel, Bao Tram Nghiem, Susan Garfield, Langston Lim, Poonam Mannan, Ana I. Robles, William I. Smith, Joshua Zimmerberg, Rea Ravin, Lalage M. Wakefield

**Affiliations:** 1 Laboratory of Cancer Biology and Genetics, National Cancer Institute, Bethesda, MD 20892, USA; 2 Protein Expression Laboratory, Advanced Technology Program, Frederick National Laboratory for Cancer Research, Frederick, MD 21701, USA; 3 Confocal Microscopy Core, National Cancer Institute, Bethesda, MD 20892, USA; 4 Laboratory of Human Carcinogenesis, National Cancer Institute, Bethesda, MD 20892 USA; 5 Department of Pathology, Suburban Hospital, Bethesda, MD 20814, USA; 6 Program in Physical Biology, National Institute of Child Health and Human Development, Bethesda, MD 20892, USA

## Abstract

Many tumors are hierarchically organized with a minority cell population that has stem-like properties and enhanced ability to initiate tumorigenesis and drive therapeutic relapse. These cancer stem cells (CSCs) are typically identified by complex combinations of cell-surface markers that differ among tumor types. Here, we developed a flexible lentiviral-based reporter system that allows direct visualization of CSCs based on functional properties. The reporter responds to the core stem cell transcription factors OCT4 and SOX2, with further selectivity and kinetic resolution coming from use of a proteasome-targeting degron. Cancer cells marked by this reporter have the expected properties of self-renewal, generation of heterogeneous offspring, high tumor- and metastasis-initiating activity, and resistance to chemotherapeutics. With this approach, the spatial distribution of CSCs can be assessed in settings that retain microenvironmental and structural cues, and CSC plasticity and response to therapeutics can be monitored in real time.

## Highlights

• A fluorescent reporter was generated that reports on the presence of SOX2 and OCT4
• Breast cancer cells marked by the reporter have characteristics of cancer stem cells
• The reporter identifies a therapy-resistant subpopulation of cancer cells
• The reporter allows visualization of cancer stem cells in vivo and in real time

## Graphical Abstract

**Figure:**
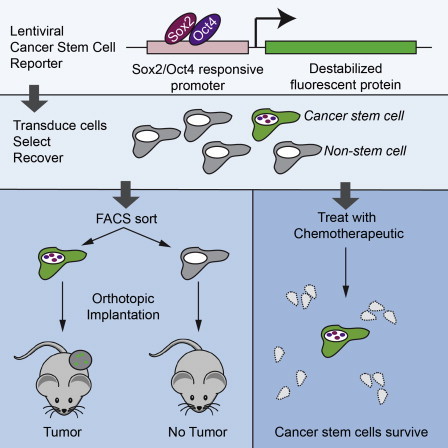

## Teaser

In this article, Wakefield and colleagues show that a subpopulation of cancer cells with characteristics of cancer stem cells can be identified and visualized in vitro and in vivo using a lentiviral-based fluorescent reporter that responds to the presence of the stemness master transcription factors SOX2 and OCT4.

## Introduction

The cancer stem cell model proposes that the parenchymal cells of tumors are hierarchically organized (Clevers, 2011; Magee et al., 2012). At the apex of the hierarchy are cells that are uniquely capable of initiating and sustaining tumorigenesis, a property that is tightly linked to their ability to self-renew. These are the cancer stem cells (CSCs), which give rise to the phenotypically diverse and more differentiated, but nontumorigenic, offspring that make up the bulk of the tumor. Thus, cancer can be viewed as a caricature of normal development (Pierce and Speers, 1988). With some notable exceptions, such as melanoma, there is evidence supporting this model for many tumor types (Magee et al., 2012), and a hierarchical structure is even maintained to some extent in established tumor cell lines cultured in vitro (Locke et al., 2005).

CSCs are thought to play a major role in driving disease recurrence, due to the intrinsically enhanced therapeutic resistance that results from high expression of multidrug transporters, enhanced DNA damage checkpoint activation and repair mechanisms, and altered cell-cycle kinetics in CSCs (Alison et al., 2012). Thus, understanding CSC biology will be critical to the development of more effective cancer therapies. CSCs are most commonly identified by fluorescence-activated cell sorting (FACS) analysis, through combinations of cell-surface markers that enrich for cell populations with enhanced tumor-initiating activity in vivo (Magee et al., 2012). However, the optimal marker combinations are very dependent on the tissue and specific cell of origin of the tumor, and even well-established markers such as CD44^+^CD24^−/lo^ for breast cancer and CD133^+^ for brain tumors do not robustly distinguish tumorigenic from nontumorigenic cells in all patient samples (Magee et al., 2012; Visvader and Lindeman, 2012). Importantly, identification of CSCs by cell-surface marker phenotype cannot readily be used to monitor CSCs in situ in the tumor, with all the extrinsic microenvironmental cues intact. Furthermore, this approach cannot be used for real-time assessment of CSC behavior at a single-cell rather than a population level. These limitations have impeded characterization of CSCs in preclinical models, where the ability to observe the CSC directly, and monitor the behavior of individual cells in time and space, would give new insights into CSCs properties and their response to therapy.

To address this need, we have developed a functional imaging approach for CSC identification. The stem cell phenotype in embryonic stem cells (ESCs) is maintained by a central triad of master transcriptional regulators, OCT4, SOX2, and NANOG, which promote stemness by upregulating genes involved in pluripotency and self-renewal while suppressing genes involved in differentiation (Young, 2011). Indeed, ectopic expression of just three factors, OCT4, SOX2, and KLF4, is sufficient to induce pluripotency and stem-like characteristics in differentiated somatic cells (Schmidt and Plath, 2012), suggesting that reactivation of stem cell transcription factors might be an efficient mechanism for transformed cells to acquire the ability to self-renew. We therefore hypothesized that OCT4 and SOX2, the two most upstream regulators of the stem cell phenotype, would be active in CSCs and could be used to drive a reporter construct that would mark the CSCs. In support of this hypothesis, embryonic stem-like gene expression signatures are found to be enriched in many aggressive tumors (Ben-Porath et al., 2008), and myeloid leukemia stem cells have been shown to employ a transcriptional program that is more similar to embryonic than adult stem cells (Somervaille et al., 2009). Promoter-reporter constructs based on portions of the promoters of *OCT4*, *SOX2*, or *NANOG* have been widely used in monitoring the reprogramming of somatic cells to the induced pluripotent state (Hotta et al., 2009) but have had only limited application in identifying CSCs (Levings et al., 2009), where expression levels of these transcription factors are likely to be much lower. In addition, the relatively large promoter regions used in such constructs invariably contain response elements for additional transcription factors, which may reduce reporter specificity.

To overcome these problems of sensitivity and specificity, we have generated a flexible, lentiviral-based stem-cell reporter system in which six tandem repeats of a composite OCT4/SOX2 response element are used to drive expression of a fluorescent protein reporter. We show that this reporter identifies a cell population in human breast cancer cell lines and primary human tumor samples that has the expected characteristics of CSCs, including enrichment for tumor-initiating ability and increased resistance to chemotherapeutics in vitro and in vivo. With this approach, the CSCs can be directly imaged in tumors and monitored by time-lapse photography for properties such as phenotypic plasticity and response to therapeutics.

## Results

### The SORE6 Reporter Marks a Minority Tumor Cell Population that Is Enriched for Stem Cell Transcription Factors

**Figure 1:**
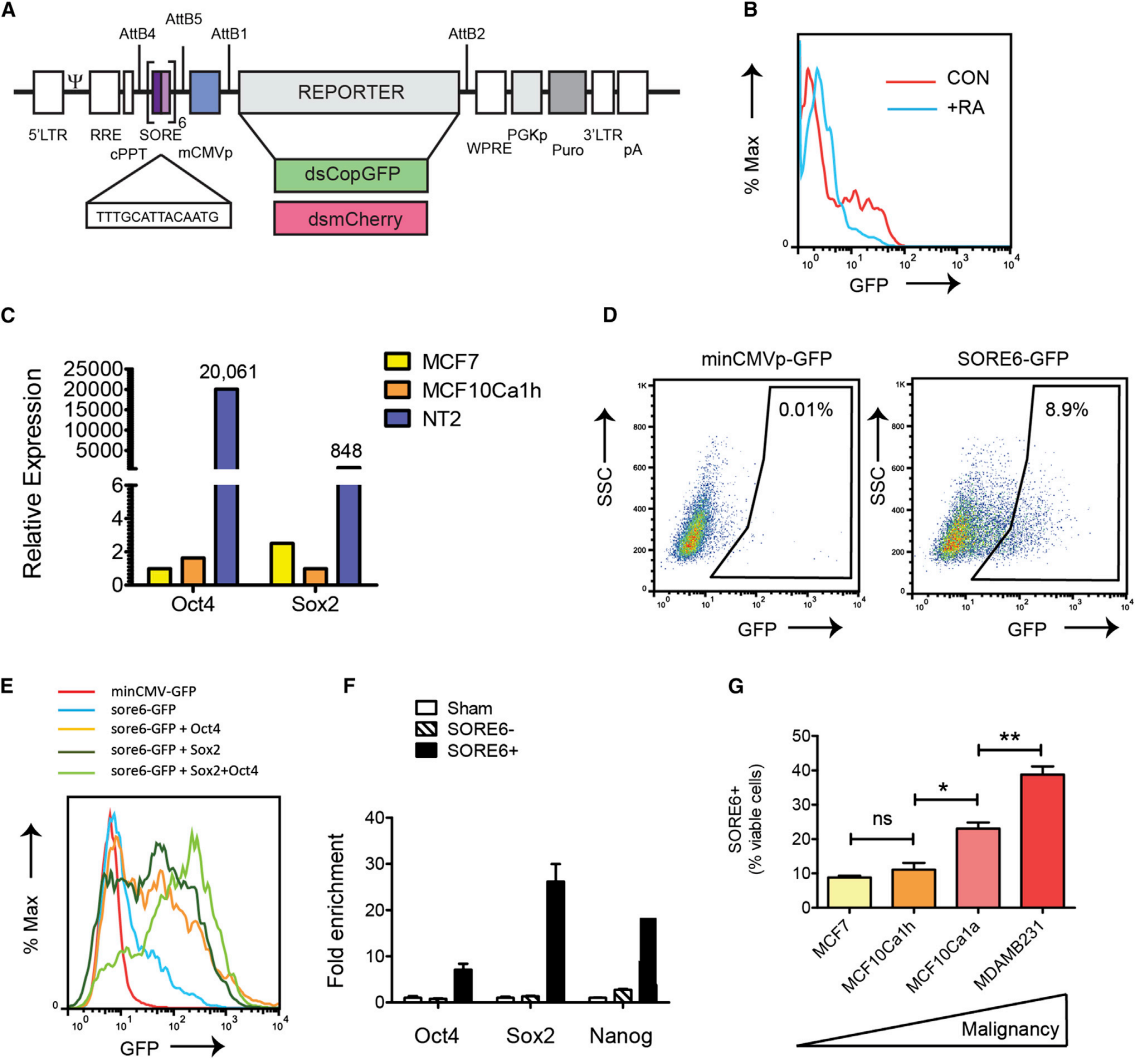
The SORE6 Reporter Marks a Minority Cell Population that Is Enriched for Stem Cell Genes (A) Schematic of the lentiviral stem cell reporter. AttB1,B2,B4,B5 represent AttB sites for modular Gateway recombinational cloning. SORE is the SOX2/OCT4 composite response element. For details of other elements, see Supplemental Experimental Procedures. (B) FACS analysis showing activity of SORE6-GFP reporter in mouse ESCs with and without treatment with retinoic acid (RA) for 2 days to induce differentiation. (C) Quantitative RT-PCR assessing the expression of stem cell transcription factors in bulk culture of breast cancer cell lines, compared with the human teratocarcinoma line NT2 as a positive control. Results are normalized to PPIA and to the lowest-expressing cell line for each gene. (D) FACS analysis showing that the SORE6 reporter identifies a minority population in cultures of MCF10Ca1h cells. SSC, side scatter. (E) FACS analysis showing effect on SORE6 reporter activity of overexpressing OCT4 and/or SOX2 in MCF10Ca1h cells. (F) Quantitative RT-PCR to assess expression of master stem cell transcription factors in FACS-sorted SORE6^+^ and SORE6^−^ cells, normalized to sham-sorted cells as the control. Results are mean ± SEM (n = 3 technical replicates). (G) Representation of SORE6^+^ cells in breast cancer cell lines of increasing malignancy. Results are mean ± SEM of three independent experiments. ^∗^p < 0.05; ^∗∗^p < 0.01; ^∗∗∗∗^p < 0.0001, Student’s t test. See also Figure S1.

We designed a modular lentiviral reporter construct in which six concatenated repeats of a composite SOX2/OCT4 response element (SORE6) from the proximal human *NANOG* promoter (Kuroda et al., 2005) were coupled to a minimal cytomegalovirus (CMV) promoter and used to drive expression of reporter genes (Figure 1A). The construct was designed using flexible Gateway multisite recombinational cloning, so that a variety of different fluorescent proteins or other genes of interest can rapidly be introduced into the construct if Att1-2 entry clones are available. The majority of our experiments used a destabilized copepod GFP-based reporter construct (SORE6-GFP), in which the destabilization of the fluorescent reporter is predicted to result in greater temporal resolution. Furthermore, since stem cells have lower-than-normal 26S proteasomal activity (Vlashi et al., 2009), the destabilization sequence adds further specificity for the stem cell. Where indicated, we used destabilized mCherry as an alternative reporter in cells that already constitutively expressed GFP.

To validate the approach, we first introduced the SORE6-GFP reporter into mouse embryonic stem cells (mESCs), which express SOX2 and OCT4 at high levels (Young, 2011). Although transduction efficiency was not high in these unselected cultures, a significant fraction of the mESCs showed strong expression of the reporter, which was greatly reduced by 2 days of treatment with retinoic acid to induce mESC differentiation (Figure 1B). Thus the reporter behaved as expected in ESCs. We then showed that two commonly used human breast cancer cell lines (MCF7 and MCF10Ca1h) express detectable levels of *SOX2* and *OCT4* mRNA in bulk culture, though the level was two to four orders of magnitude lower than is seen in the human teratocarcinoma line NT2 (Figure 1C). It should be noted that the *OCT4* primer pair we used does not detect the *OCT4* pseudogenes that can confound this type of analysis (Atlasi et al., 2008; Lengner et al., 2008).

To determine whether such low levels of SOX2 and OCT4 were sufficient to drive reporter expression, we transduced the MCF10Ca1h breast cancer cell line with the SORE6 reporter. Following selection with puromycin to ensure the presence of reporter construct in all cells, we found the SORE6-GFP reporter to be expressed in a minority population of cells in the culture (SORE6^+^ cells), ranging from ∼7%–15% depending on culture conditions (Figure 1D). A construct with the minimal CMV promoter, but lacking the SORE6 element, was used as a gating control. Experimental overexpression of SOX2 and OCT4 in the MCF10CA1h cells showed that the reporter can respond to either factor individually, but strongest expression is seen when both are present (Figure 1E). Conversely, simultaneous knockdown of endogenous SOX2 and OCT4 with small interfering RNA (siRNA) gave a greater reduction in reporter expression than knockdown of either individually (Figures S1A and S1B available online). On a single-cell level, all cells that were positive for the SORE6-GFP reporter expressed OCT4 (Figure S1C). As expected, SORE6+ cells recovered by FACS sorting from MCF10Ca1h cells transduced with SORE6-GFP showed substantial enrichment (7- to 26-fold) for transcripts of the core stem cell transcription factors *OCT4*, *SOX2*, and their downstream target *NANOG* (Figure 1F). We next compared expression of the reporter in several breast cancer cell lines representing different degrees of malignancy. The relatively well-differentiated, estrogen-receptor-positive breast cancer cell lines MCF7 and MCF10Ca1h had ∼10% SORE6^+^ cells, while the more malignant MCF10Ca1a and the highly aggressive MDA-MB-231 cells had an increasingly higher representation of SORE6^+^ cells in the culture (Figure 1G). *SOX2* and *OCT4* mRNA levels were correspondingly higher in the more malignant cell lines (Figure S1D). Thus, despite the low expression of stem cell transcription factors in bulk culture, the SORE6 reporter is capable of identifying a minority population of cells that express these factors in several breast cancer cell lines.

### The SORE6^+^ Population Can Self-Renew, Give Rise to Phenotypically Heterogeneous Offspring, Divide Asymmetrically, and Form Tumorspheres In Vitro

**Figure 2:**
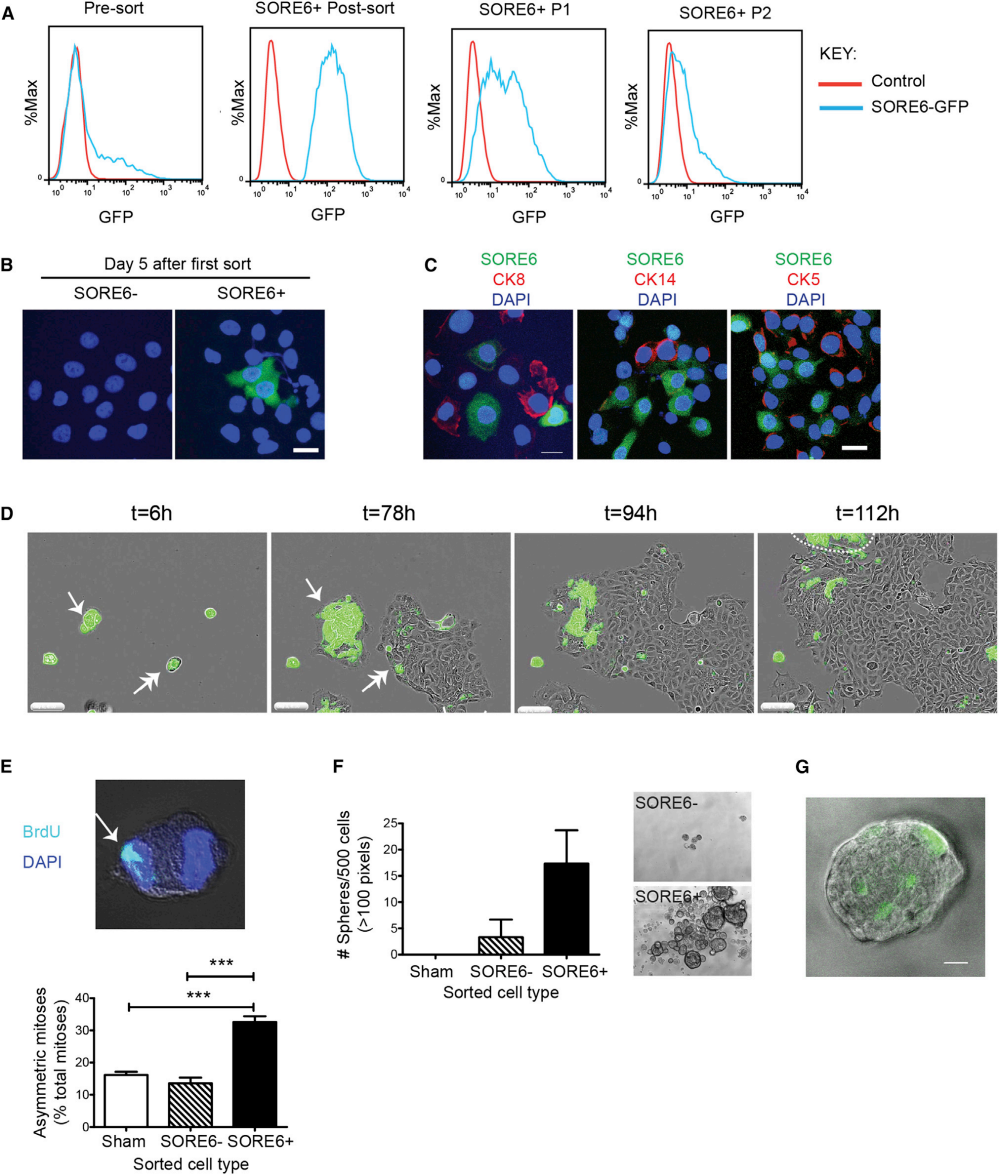
SORE6^+^ Cells Are Enriched for the Ability to Self-Renew, Generate Heterogeneous Offspring, Undergo Asymmetric Division, and Generate Tumorspheres (A) FACS plots showing that sorted SORE6^+^ MCF10Ca1h cells can regenerate SORE6^−^ cells in culture. P1, first passage after sort; P2, second passage. (B) Fluorescent images showing sorted SORE6^+^ or SORE6^−^ MCF10Ca1h cells after 5 days in culture. Cell nuclei are visualized with DAPI (blue), and SORE6^+^ cells are green. (C) MCF10Ca1h culture from (B) immunostained for cytokeratin 5 (CK5), cytokeratin 8 (CK8), or cytokeratin 14 (CK14). Scale bar, 20 μm. (D) Freeze frames from the time-lapse Movie S1 showing SORE6^+^ cells generating SORE6− offspring. MCF10Ca1h cultures enriched for SORE6^+^ cells followed by time-lapse videomicroscopy. In frame 1 (t = 6 hr), the single-headed arrow marks a small cluster of SORE6^+^ cells (cluster 1) and the double-headed arrow marks a doublet of SORE6^+^ and SORE6^−^ cells (cluster 2). Frame 2 (t = 78 hr) shows that cluster 1, after undergoing several symmetric self-renewing divisions, has begun to generate SORE6^−^ cells around the periphery of the colony. Cluster 2 has now generated a colony on the right that is predominantly SORE6^−^, suggesting the SORE6^−^ cells may proliferate faster than the SORE6^+^ cells. Frame 3 (t = 94 hr) and frame 4 (t = 112 hr) show that when cluster 2 expands to contact cluster 1, there is a rapid loss of SORE6^+^ cells in cluster 1. The group of predominantly SORE6^+^ cells marked by the dashed line in the top left of frame 4 has migrated in from outside of the field. Scale bar, 200 μm. (E) Asymmetric mitoses in FACS sorted SORE6^+^ and SORE6^−^ or sham-sorted MCF10Ca1h cultures. Representative z stack image of asymmetrically distributed BrdU-labeled DNA in a pair of mitotic daughter cells and quantitation of asymmetric mitoses as % total mitoses. Results are mean ± SEM for three independent experiments, each evaluating 30–50 mitoses/condition. ^∗∗∗^p < 0.001; Student’s t test. (F) Tumorsphere formation by sorted SORE6^+^ and SORE6^−^ or sham-sorted MCF10Ca1h cells. Results are mean ± SEM (three independent experiments). Representative phase-contrast images of tumorspheres are shown. (G) Fluorescent image of large tumorsphere derived from sorted SORE6^+^ MCF10Ca1h cells. Scale bar, 20 μm. See also Figures S2 and S3 and Movie S1.

A central tenet of the CSC hypothesis is that CSCs can self-renew and give rise to more committed daughter cells, while the regeneration of CSCs from more differentiated daughter cells is a much lower frequency event (Magee et al., 2012). To address this issue, MCF10Ca1h cells were sorted into SORE6^+^ and SORE6^−^ populations and placed in culture. The SORE6^+^ population rapidly regenerated a SORE6^−^ cell population, which increased with passage in culture until the original equilibrium state was restored by passage ∼2–3 (Figures 2A and 2B). In contrast, SORE6− cells were largely incapable of regenerating a SORE6^+^ population (Figure 2B). MCF10Ca1h tumors have differentiated luminal and myoepithelial components, consistent with the CSC having arisen from a bipotential progenitor (Santner et al., 2001). As expected, the SORE6^−^ daughters arising from SORE6^+^ cultures expressed the differentiated luminal marker cytokeratin 8 (CK8) or the basal marker cytokeratin 14 (CK14), while the SORE6^+^ cells were negative for these markers but showed some positivity for CK5 (Figure 2C), a marker of more primitive progenitors (Kabos et al., 2011). Thus, the SORE6^+^ cells themselves are relatively undifferentiated but can give rise to differentiated offspring of both mammary epithelial lineages. Time-lapse videomicroscopy clearly showed the appearance of SORE6^−^ cells in colonies that grew from SORE6^+^ cells (Figure 2D; Movie S1).

In somatic stem cells, self-renewal is often associated with the ability to undergo asymmetric cell divisions, in which one daughter cell retains the property of stemness, while the other is committed to differentiate (Magee et al., 2012). Asymmetric division with respect to cell fate can involve asymmetric segregation of newly synthesized DNA strands (Conboy et al., 2007), and this type of asymmetric division has been phenotypically associated with a hierarchical organization and cell fate in lung cancer models (Pine et al., 2010). By assessing the frequency of asymmetric distribution of bromodeoxyuridine (BrdU)-labeled chromatin between mitotic daughters, we showed that SORE6^+^ cells had a higher frequency of asymmetric division than SORE6^−^ or sham-sorted cultures (Figure 2E). Another property of normal and malignant stem cells is the ability to proliferate and form large sphere-like structures under anchorage-independent conditions (Shaw et al., 2012). We showed that SORE6^+^ cells from MCF10Ca1h cultures were enriched for the ability to form large tumorspheres (Figure 2F) and that each tumorsphere contained just one or a few SORE6^+^ cells (Figure 2G), consistent with previous observations that such spheres contain an average of one sphere-forming cell (Shaw et al., 2012). Proteasomal blockade with MG-132 to slow degradation of the destabilized GFP reporter moiety led to an ∼2-fold increase in the proportion of SORE6^+^ cells in MCF7 cultures and a corresponding decrease in tumorsphere-forming efficiency of the SORE6^+^ fraction (Figure S2), confirming that the destabilizing sequence on the GFP significantly increases the specificity of the reporter. Overall, the data show that the SORE6 reporter marks cells that are relatively undifferentiated, with the ability to self-renew, divide asymmetrically, and give rise to phenotypically heterogeneous, more differentiated offspring, all of which are important properties of CSCs.

Breast cancer CSCs have been identified by cell-surface marker combinations, most commonly CD44^+^CD24^lo/−^, as well as by expression of ALDH1 (Visvader and Lindeman, 2012), so we investigated the status of these CSC markers in our SORE6^+^ population. We found no enrichment of the CD44^+^CD24^−^ marker combination in our SORE6^+^ fractions and substantial though variable overlap with the ALDH1-positive population (Figure S3). The overlap between the CD44^+^CD24^−^ marker combination and ALDH positivity has previously been shown to be very low (Ginestier et al., 2007), suggesting that existing methods for detecting CSCs are not fully concordant. Furthermore, the CD44^+^CD24^−^ phenotype correlated more closely with basal phenotype than with tumorigenicity in breast cancer cell lines (Fillmore and Kuperwasser, 2008). It is becoming apparent that there is heterogeneity even within stem cell populations (Schober and Fuchs, 2011), so it is possible that the different methods enrich different subpopulations of CSCs.

### SORE6^+^ Cells Are Enriched for Tumor- and Metastasis-Initiating Activity In Vivo

**Figure 3:**
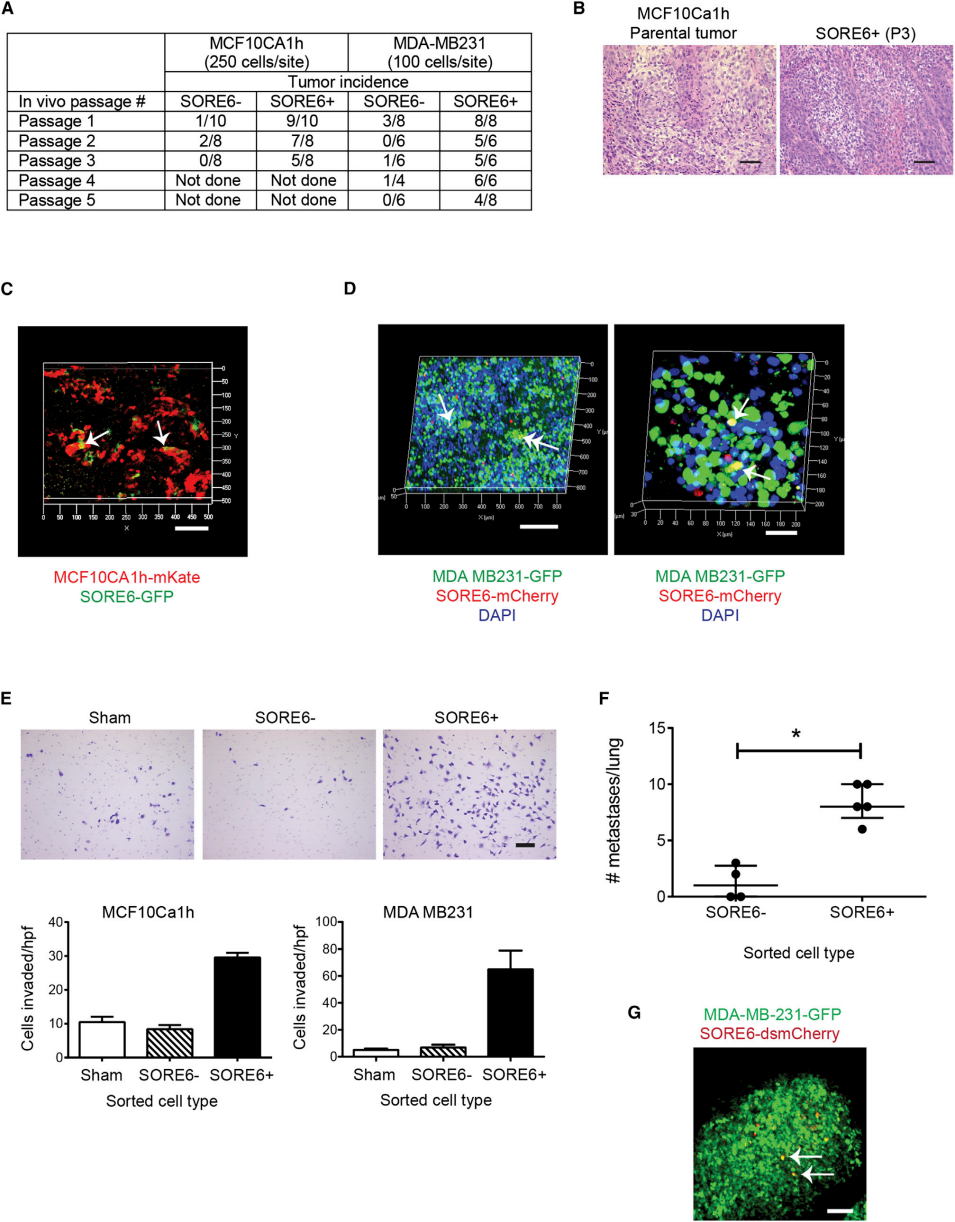
SORE6^+^ Cells Are Enriched for Tumor- and Metastasis-Initiating Ability In Vivo and Can Be Visualized In Situ (A) Tumor-initiating ability of SORE6^+^ cells is maintained over multiple transplant generations. (B) H&E-stained sections showing histology of parental MCF10Ca1h tumors and tumors generated by SORE6^+^ cells after three serial passages in vivo. Scale bar, 50 μm. (C) Confocal z stack image showing spatial localization of SORE6^+^ cells in freshly excised MCF10Ca1h tumors. Tumor cells are constitutively marked in red, and SORE6^+^ cells are green. Arrows point to SORE6^+^ cells. Scale bar, 100 μm. (D) Confocal z stack image showing spatial localization of SORE6^+^ cells in MDA-MB-231 tumors. Note that here, tumor cells are constitutively marked in green, while SORE6^+^ cells are red. Arrows indicate yellow SORE6^+^ tumor cells. In this image, the red dots are not associated with nuclei and probably represent dead cell debris. Scale bar, 200 μm (left) or 40 μm (right). (E) Matrigel invasion assays using sorted SORE6^+^ and SORE6^−^ and sham-sorted cells from MCF10Ca1h and MDA-MB-231 cultures. Results are mean ± SEM (n = 3 technical replicates). Representative images are shown for the MDA-MB-231 cells. Scale bar, 100 μm. (F) Lung metastases formed following tail-vein injection of sorted SORE6^+^ and SORE6^−^ and sham-sorted cells from MDA-MB-231 cultures. Results are shown as median ± interquartile range for n = 5 mice/group. ^∗^p < 0.05, two-way ANOVA. (G) Confocal z stack image of a lung metastasis derived from a SORE6^+^ MDA-MB-231 cell, showing rare yellow cells (some marked by arrows) that are positive for the SORE6 reporter. The tumor cells are constitutively marked with GFP, while the SORE6 reporter drives dsmCherry. Scale bar, 200 μm.

The gold-standard assay for a CSC is the ability to initiate and sustain tumorigenesis in vivo. We performed an in vivo limiting dilution assay in the MCF10Ca1h model to assess the relative tumor-initiating ability of SORE6^+^ and SORE6^−^ cells following orthotopic implantation into nude mice, and we observed a ∼20× enrichment of tumor initiating capacity in the SORE6^+^ compared with the SORE6^−^ cell populations (Table 1). Similar enrichment was seen with two additional breast cancer models, MCF7 cells (estrogen-receptor-positive breast cancer) and MDA-MB-231 cells (triple-negative breast cancer) (Table 2). To assess long-term self-renewal and tumor-initiating ability, cells were recovered from tumors that arose at each passage and were resorted into SORE6^+^ and SORE6^−^ fractions and reimplanted for the subsequent serial in vivo passage. SORE6^+^ cells sustained the ability to initiate tumorigenesis through multiple serial transplant generations in both the MCF10Ca1h and MDA-MB-231 models (Figure 3A). MCF10Ca1h tumors characteristically show a heterogeneous histology with areas of clear cells along with well differentiated structures and areas of poorly differentiated pleomorphic cells (Santner et al., 2001; Tang et al., 2003), and tumors derived from MCF10Ca1h SORE6^+^ cells after three serial transplant generations showed the same histopathology as the parental cell line (Figure 3B). The occasional small tumors that arose from implantation of SORE6^neg^ Lin^neg^ cells were invariably found to contain a small population (0.2%–0.3%) of SORE6^+^ cells, suggesting either that the FACS sort was not 100% efficient or that SORE6^+^ cells may be generated from SORE6^−^ cells as a low-frequency event in vivo. Confocal images of MCF10Ca1h tumors confirmed that the SORE6^+^ cells were a minority population in vivo and showed individual SORE6^+^ cells or small clusters of SORE6^+^ cells scattered through the tumor parenchyma (Figure 3C). A similar pattern was seen in MDA-MB-231 tumors, where the CSCs tended to be localized in clusters (Figure 3D). Note that for the MDA-MB-231 model, the stem cell reporter is red (SORE6-dsmCherry), since the tumor cells were already constitutively marked with GFP.

It has been proposed that a subset of CSCs may be intrinsically migratory and/or invasive (Brabletz et al., 2005). Using a Matrigel invasion assay, we found that SORE6^+^ cells from the nonmetastatic MCF10CA1h and metastatic MDA-MB-231 cell lines were significantly more invasive than SORE6^−^ or sham-sorted cells (Figure 3E). Furthermore, SORE6^+^ cells were strongly enriched for the ability to initiate metastases in the lung in vivo following injection into the tail vein (Figure 3F). Individual metastases that formed from SORE6^+^ cells showed just a small fraction of SORE6^+^ cells, with the bulk of the cells in the lesion having differentiated to a SORE6^−^ phenotype (Figure 3G). As was seen with the primary tumors, the rare metastases that formed from SORE6^−^ cells also showed the presence of SORE6^+^ cells, reflecting either incomplete sorting or phenotypic plasticity. In either case, it appears that the development of a metastasis is invariably associated with the presence of SORE6^+^ cells.

### SORE6+ Cells Are Relatively Resistant to Chemotherapeutics

**Figure 4:**
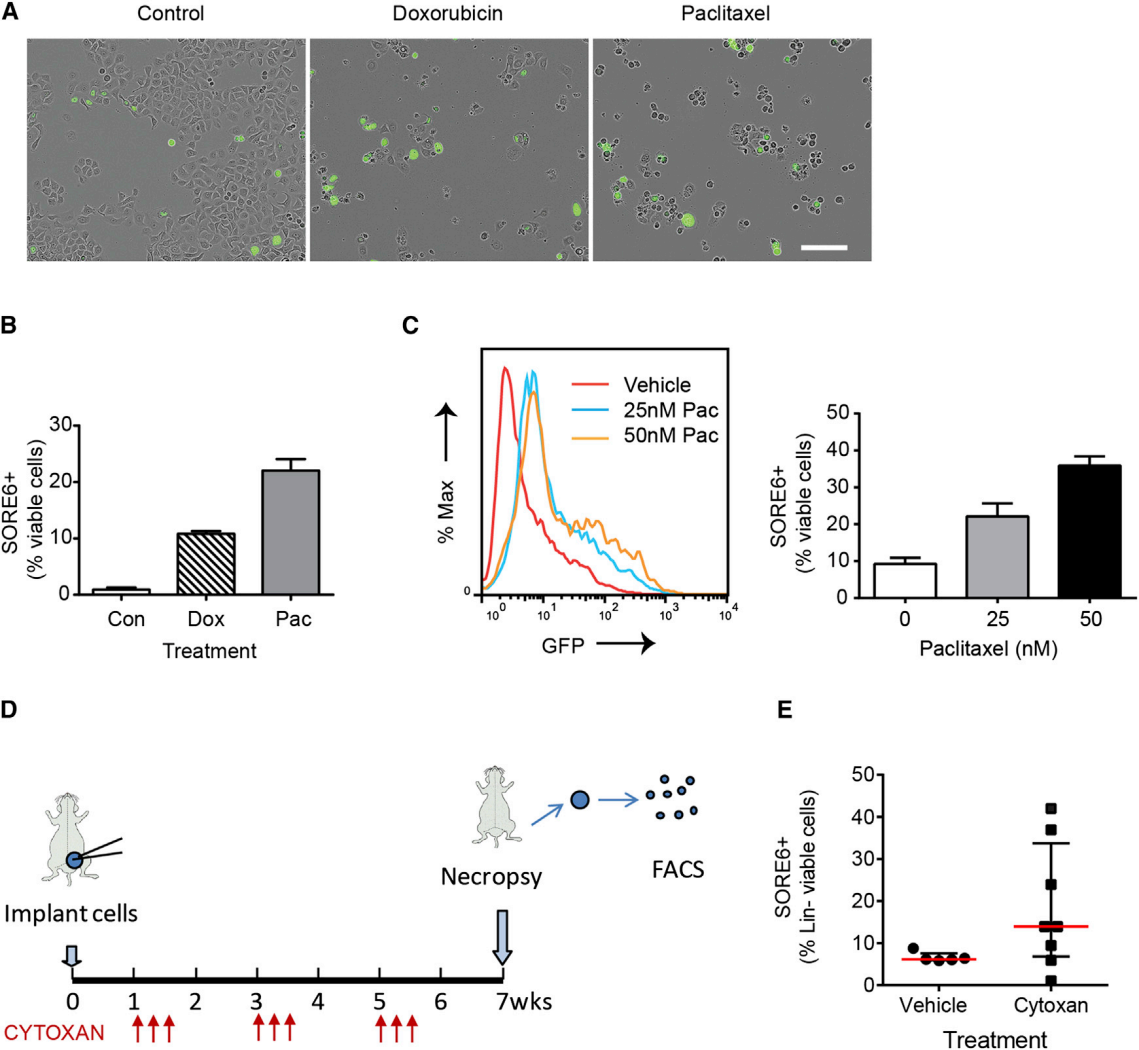
SORE6^+^ Cells Are Relatively Resistant to Chemotherapeutics (A) Cultures of MCF10Ca1h cells after 2 days of treatment with doxorubicin (50 nM) or paclitaxel (25 nM) showing selective killing of SORE6^−^ cells. See also Movies S2 and S3. Scale bar, 200 μm. (B) Effect of treatment with doxorubicin (Dox; 50 nM) or paclitaxel (Pac; 25 nM) on the relative representation of SORE6^+^ cells in the MCF10Ca1h culture assessed by flow cytometry after 48 hr. Results are mean ± SEM for three technical replicate determinations. (C) FACS profile of MCF10Ca1h cells after 4 days of treatment with 25 nM or 50 nM paclitaxel (Pac), together with quantitation of SORE6^+^ cells by FACS analysis. Results are mean ± SEM for three technical replicates. (D) Schematic for treatment of MCF10Ca1h tumors with Cytoxan. (E) The effect of Cytoxan on SORE6^+^ cell representation in tumors from Cytoxan- or vehicle-treated mice. Results are median ± interquartile range for n = 5–8 mice/group. See also Movies S2 and S3.

CSCs are intrinsically more resistant to chemotherapeutics (Alison et al., 2012). On treatment of MCF10Ca1h cultures with doxorubicin (50 nM) or paclitaxel (25 nM) for 2 days, extensive cell death was observed among SORE6- cells (Figure 4A; Movies S2 and S3), and the proportion of SORE6^+^ cells in the culture increased dramatically (Figure 4B). The effect of paclitaxel was dose dependent, with greater enrichment of SORE6^+^ cells at higher doses (Figure 4C). Similar results were seen in vivo, where treatment of mice bearing MCF10Ca1h tumors with the chemotherapeutic Cytoxan led to a substantial increase in the proportion of SORE6^+^ cells in the tumors after three cycles of treatment (Figures 4D and 4E).

### The SORE6 Reporter Marks a Minority Population of Cells with Tumor-Initiating Activity in Primary Human Tumor Cell Cultures

**Figure 5:**
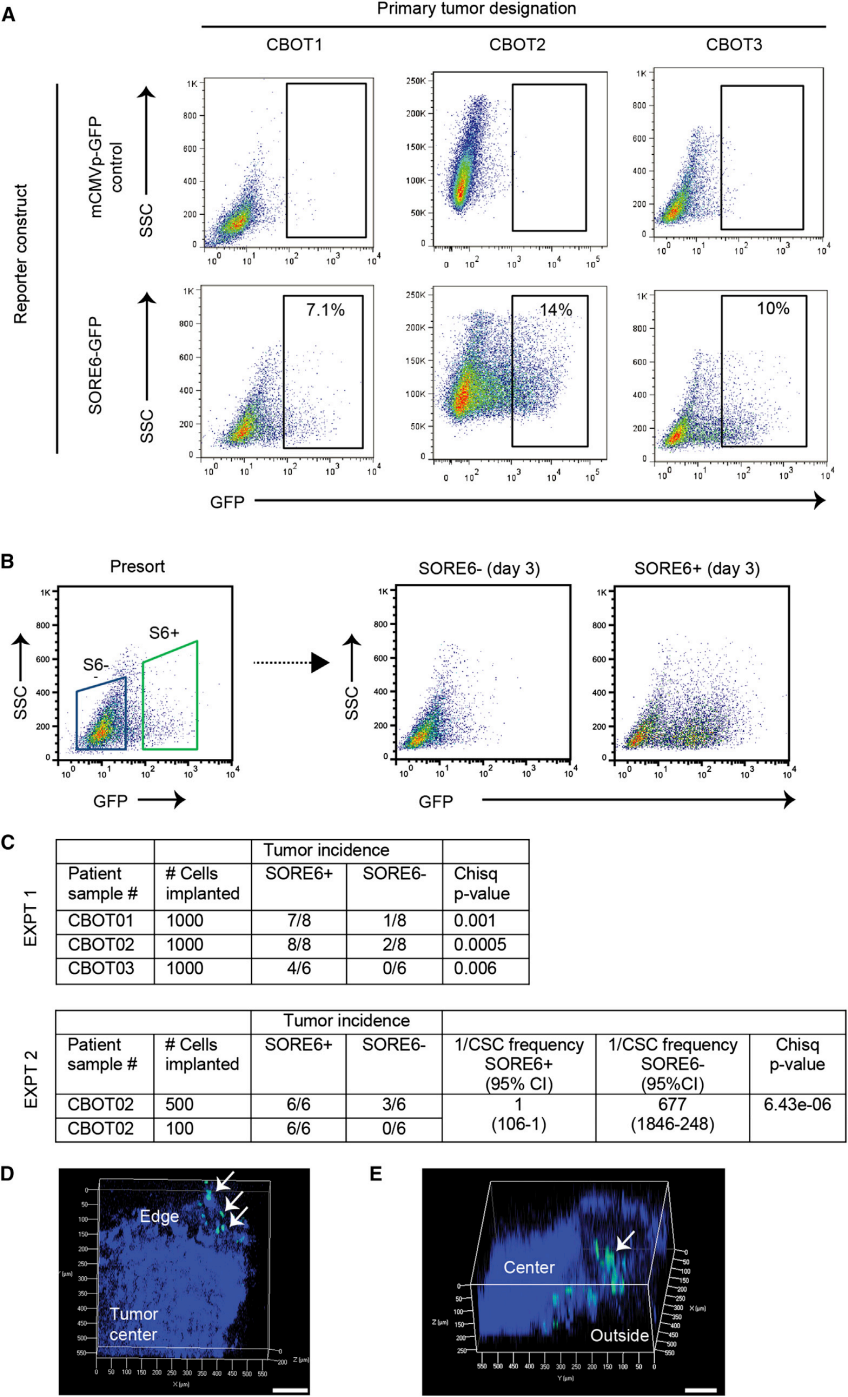
The SORE6 Reporter Marks a Minority Population with Enhanced Tumor-Initiating Activity in Primary Cultures from Human Breast Cancer (A) Explant cultures of three independent primary human breast cancers were transduced with the SORE6-GFP reporter or minCMVp-GFP control lentivirus, and the SORE6^+^ population was assessed by FACS. (B) FACS plots showing that SORE6^+^ cells from the primary human breast cancer culture CBOT1 can regenerate significant numbers of SORE6^−^ cells after 3 days in culture. (C) Relative enrichment of SORE6^+^ cells for tumor-initiating ability in the primary human breast cancer cultures, assessed by implantation of sorted cells in vivo. Two independent experiments were performed. In experiment #2, stem cell frequencies were calculated using ELDA software. (D) Confocal z stack image (50 μm depth) of freshly excised xenografted tumor derived from SORE6^+^ primary human breast cancer cells showing green SORE6^+^ cells in clusters at the edge of the tumor (arrows). Blue represents DAPI-stained nuclei. Scale bar, 100 μm. (E) Deeper (250 μm) confocal z stack image showing SORE6^+^ cells (arrow) at edge of tumor. Blue indicates tumor as visualized by second harmonic generation, since DAPI could not penetrate to sufficient depth. Scale bar, 100 μm.

All the experiments to this point were done with well-established human breast cancer cell lines. To test whether the reporter could be used to transduce primary tumor cell cultures, we acquired eight primary human breast cancer samples of which three successfully generated explant cultures and patient-derived xenografts. Explanted tumor cell cultures were transduced with SORE6 or minCMVp control reporters and briefly selected with puromycin. As with the cell lines, a minority of cells (7%–14%) in the primary cultures were SORE6^+^ (Figure 5A). Sorted SORE6^+^ cells placed in culture regenerated a significant population of SORE6^−^ cells within 3 days, while the SORE6^−^ cells failed to regenerate SORE6^+^ cells (Figure 5B). On implantation in athymic nude mice, the sorted SORE6^+^ cells were significantly more tumorigenic than SORE6^−^ cells for all three primary samples (Figure 5C). Finally, confocal images of freshly excised xenografted CBOT01 tumors arising from SORE6^+^ cells show clusters of SORE6^+^ cells localized primarily at the edge of the tumor (Figures 5D and 5E). Overall, the data suggest that the SORE6 reporter can identify a subpopulation of tumor cells that are enriched for CSC-like properties in primary cultures of human breast cancer as well as in established cell lines.

## Discussion

It is increasingly appreciated that a tumor represents a whole ecosystem of mutually interacting cellular and acellular components that generate a continually evolving tumor microenvironment (Quail and Joyce, 2013). Many aspects of this dynamic and complex microenvironment, such as hypoxia and inflammation, can modulate CSC properties and response to therapy (Conley et al., 2012; Cui et al., 2013; Korkaya et al., 2012) and function in different spatial contexts within the tumor. Thus, it would be desirable to observe the behavior of the CSCs in their native habitat with all microenvironmental cues intact. Here, we have developed and validated a flexible and powerful lentiviral-based reporter system for direct visualization, quantitation, and isolation of the cells with CSC properties in multiple preclinical tumor models in vitro and in vivo. Cells detected by this method are relatively undifferentiated, can self-renew and give rise to phenotypically heterogeneous offspring, show enhanced asymmetric division, and are enriched for tumor-initiating and metastasis-initiating ability in vivo. Importantly, the marked cells are also relatively resistant to chemotherapeutics, suggesting that a highly clinically relevant tumor cell subpopulation is being detected with this reporter.

Our approach depends on the presence of the stemness transcription factors SOX2 and/or OCT4 in the CSC. SOX2 is expressed in immature cells of many self-renewing epithelial tissues in the adult animal (Arnold et al., 2011), and it has been detected in a variable percentage of cells in many malignant tissues, some of which clearly depend on SOX2 for their tumor-initiating ability (Gangemi et al., 2009). Detection of *OCT4* is complicated by the existence of alternate transcripts and pseudogenes, and evidence is convincing that *OCT4* is not expressed in adult somatic stem cells (Lengner et al., 2008). However, ectopic expression of OCT4 in the intestinal epithelium and epidermis blocks differentiation and leads to uncontrolled proliferation of progenitor cells (Hochedlinger et al., 2005), and forced overexpression of OCT4 in primary breast epithelial cells generated tumor-initiating cells (Beltran et al., 2011), suggesting that reactivation of epigenetically silenced *OCT4* would be a parsimonious route to tumor formation. Ionizing radiation was recently shown to reprogram differentiated breast cancer cells into cells with CSC characteristics associated with reexpression of OCT4 and SOX2, further supporting an intimate connection between stemness and OCT4/SOX2 expression (Lagadec et al., 2012). So far, our reporter has identified a minority cell population in all the primary and established breast cancer cells we have studied, suggesting that the presence of functional SOX2/OCT4 in a subpopulation of tumor cells may be a relatively widespread phenomenon.

Complementary approaches to visualizing the CSCs have taken advantage of different biological properties of the tumor hierarchy, such as low expression of 26S proteasome activity in CSCs (Vlashi et al., 2009) or high expression of *LET7C* in differentiated cells (Ibarra et al., 2007). Our construct uses a tandemly repeated OCT4/SOX2 response element to drive reporter expression, but our fluorescent protein also incorporates the ornithine decarboxylase degron sequence that is targeted by the 26S proteasome (Li et al., 1998), and this feature confers additional specificity for the CSCs. Despite the relatively low expression of SOX2 and OCT4 in CSCs, we have shown that our reporter can be used to detect and localize CSCs in freshly excised tumors and metastasis-bearing lungs. In principle, it should be possible to extend the approach to intravital imaging. Such a strategy will allow further investigation of the location of CSCs in different tumors, the nature of CSC niches, interactions between CSCs and their microenvironment, and longitudinal monitoring of migration and survival characteristics of CSCs, both in the unperturbed state and in response to therapeutic intervention.

Importantly, the tumor cell subpopulation marked by our reporter is considerably more resistant to conventional chemotherapy than the bulk population, and the reporter system has the potential to be adapted to a high throughput format to screen for drugs that target these resistant cells. Although the hierarchical organization of normal tissues is relatively rigid and unidirectional, there is evidence for greater plasticity in the organizational structure of tumors (Magee et al., 2012), and this plasticity will pose challenges for effective therapy if non-CSCs can reacquire CSC attributes. With our reporter system, it will be possible to observe stem cell plasticity directly, whether driven by intrinsic mechanisms, such as stochastic fluctuations in gene expression, or through extrinsic mechanisms, such as induction of an epithelial-to-mesenchymal transition (Mani et al., 2008), irradiation (Lagadec et al., 2012), or inflammation (Korkaya et al., 2011; Schwitalla et al., 2013). The ability to observe the CSCs directly and in real time as they interact with neighboring cells or environmental components should generate new insights and suggest testable hypotheses regarding the properties of this critically important cell population in many preclinical cancer model systems.

## Experimental Procedures

### Cell Culture and Treatment with Chemotherapeutics

The MCF10CA1h and MCF10Ca1a cell lines were obtained from the Karmanos Cancer Institute Cell Line Resource and cultured in Dulbecco’s modified Eagle’s medium (DMEM)/F12 with 5% horse serum (Santner et al., 2001). Early-passage MCF7 cells were obtained from Dr. Michael Brattain and were cultured in Eagle’s minimum essential medium, 10% fetal bovine serum (FBS) with 2 mM glutamine and 1% nonessential amino acids. MDA-MB-231 cells from the American Type Culture Collection (ATCC) were cultured in DMEM with 10% FBS. The mESC line R1/E from ATCC was cultured in 0.1% gelatin-coated cell culture plates with mESC growth medium containing KO-DMEM, 15% FBS, and 100 mM nonessential amino acids, 0.1 mM 2-mercaptoethanol, and 2 mM L-glutamine plus 1,000 U/ml leukemia inhibitory factor (Millipore). Differentiation of R1/E cells was induced by treatment with 5 μM retinoic acid for 4 days and confirmed by visual assessment of cell morphology. Where indicated, tumor cells were treated with 50 nM doxorubicin or 25–50 nM paclitaxel for 2 days prior to analysis by flow cytometry. Details of primary breast cancer cultures are given in Supplemental Experimental Procedures.

### Generation of Lentiviral Reporter Constructs

A stem cell enhancer minigene was designed, based on the observation that the proximal *NANOG* promoter region has highly conserved composite binding element for SOX2 and OCT4 with the sequence 5′-TTTTGCATTACAATG-3′ that is essential for properly regulated expression of NANOG in ESCs (Ibarra et al., 2007). A minigene containing six tandem repeats of this composite element with eight bases of native flanking sequence on either side of the element, was synthesized by Integrated DNA Technology and named “SORE6” for SOX2/OCT4 response element × 6. Lentiviral reporter constructs were generated by Gateway Multisite LR recombinational cloning using the manufacturer’s protocols. Individual entry clones were generated for the SORE6 minigene, a minimal CMV promoter (minCMVp), and two destablized fluorescent proteins, dscopGFP and a new destabilized form of the monomeric Cherry fluorescent protein (dsmCherry) that we constructed by addition of the PEST destabilization sequence (Corish and Tyler-Smith, 1999). Entry clones were assembled into pDest-663, a lentiviral destination vector based on the pFUGW lentiviral backbone with puromycin selection. A detailed description of minigene sequence, generation of the Entry clones and the recombinational cloning strategy is given in Supplemental Experimental Procedures. minCMVp-GFP and minCMVp-mCherry constructs in which the SORE6 element was omitted serve as matched controls to allow assessment of background expression of fluorescent proteins due to the minimal CMV promoter alone.

### Lentivirus Generation and Cell Transduction

Replication-defective infectious lentivirus was generated using the pPACK1 Lentiviral Vector Packaging Kit (Systems Biosciences). For transduction with lentiviral constructs, exponentially growing target cells were exposed to viral supernatants at an MOI of 1 for 24 hr with 5 μg/ml Polybrene. Transduction efficiency was typically >80%. Transduced cultures were either selected with 2 μg/ml puromycin for 5 days or the 5% brightest cells in the SORE6^+^ gate were collected by FACS sorting and put back in culture to recover the original population equilibrium. Transduced mouse embryonic stem cells were used without further selection since puromycin induced differentiation. In vivo experiments were performed within 2–3 weeks of transduction.

### In Vivo Tumorigenesis and Metastasis

All animal studies were done under a protocol (LC-070) approved by the National Cancer Institute, in accordance with Association for Assessment and Accreditation of Laboratory Animal Care guidelines. To determine tumor-initiating capacity in different cell populations, in vivo limiting dilution assays were performed and CSC frequency was calculated using extreme limiting dilution analysis (ELDA) (Hu and Smyth, 2009). Breast cancer cell lines or primary cultures were sorted, where applicable, and suspended in serum-free DMEM/F12 medium with 50% of growth factor reduced Matrigel (BD Bioscience), and 100–5,000 cells were surgically implanted into the #2 and #7 mammary fat pads of 6- to 8-week-old female athymic NCr nu/nu mice (Animal Production Program, Frederick National Laboratory for Cancer Research, Frederick, MD). MCF7 cells were inoculated into ovariectomized mice that had been implanted with 1.7 mg slow-release estradiol pellets (Innovative Research). Tumors were measured weekly with calipers and all mice on a given experiment were euthanized with CO_2_ before the tumor diameter of the largest tumor reached 2 cm (typically 2–3 months for MCF10Ca1h and MCF7EP tumors and 1–2 months for MDA-MB-231 tumors). To determine the metastatic potential of MDA-MB-231 cells, 5-week-old female nude mice were injected intravenously with 100,000 tumor cells in 0.2 ml of DMEM in the tail vein. The mice were euthanized 8 weeks after tumor cell inoculation, and lungs were harvested for fluorescent imaging or for histologic assessment of metastatic burden on hematoxylin and eosin (H&E)-stained sections of formalin-fixed inflated lungs.

### Cell Recovery from Xenografted Tumors

Freshly excised tumors were minced with scalpel blades, and tumor pieces were digested with DMEM/F12 medium containing 5% horse serum, 1 mg/ml collagenase I (Sigma), and 1 mg/ml collagenase D (Sigma) for 2 hr at 37°C. Cells were then washed with Hank’s balanced salt solution (HBSS) (Invitrogen) and suspended in 0.05% Trypsin/EDTA (Invitrogen) for 5 min at room temperature (RT). During trypsinization, cells were passed through 18G, 22G, 27G needles followed by passage through a 40 μm cell strainer (BD Bioscience). Following addition of Trypsin Neutralizer Solution (Invitrogen), cells were collected by brief centrifugation. Cell pellets were washed with HBSS, suspended in DMEM/F12 medium, and analyzed by flow cytometry or FACS sorted.

### Flow Cytometry and Fluorescence-Activated Cell Sorting

Subconfluent cultured cells were collected by trypsinization and cell pellets were washed three times with PBS prior to resuspension in PBS with 4% fetal bovine serum. Flow cytometry was done on a FACSCalibur (Becton Dickinson) for GFP expression alone or an LSR II (BD Biosciences) for detecting mCherry and GFP expression, and data were analyzed using FlowJo software (Tree Star). Cells transduced with the minCMVp-GFP or minCMVp-mCherry lentiviruses were used as matched negative controls for gating purposes, and cells were defined as SORE6^+^ if the fluorescence in the FL1 channel exceeded that of 99.9% of the cells transduced with control virus. For FACS, cells transduced with SORE6-GFP were sorted using a BD FACS Aria IIu Cell Sorter (BD Bioscience), while cells transduced with SORE6-mCherry were sorted using a MoFlo Astrios High Speed Sorter (Beckman Coulter). Again, minCMVp-GFP or minCMVp-mCherry were negative controls for gating, and typically, the top 5% of cells in the SORE6^+^ gate were collected. Cells recovered from tumors were stained with 20 μl /10^6^ cells of APC Mouse Lineage Antibody Cocktail (BD Bioscience) for 30 min at RT, washed with HBSS, and analyzed by flow cytometry or sorted by FACS as above. For analysis of cell-surface marker profiles, cells were labeled with allophycocyanin-conjugated-CD44 and phycoerythrin-conjugated CD24 antibodies (BD Pharmingen). For all analyses and sorts, dead cells were eliminated by 7AAD staining.

### Time-Lapse Videomicroscopy and Immunofluorescence

A total of 2,500–50,000 cells were seeded in 12-well plates. Nine images per well were acquired every 2–3 hr for a period of 2–5 days, using the IncuCyte^FLR^ live-cell imaging system (Essen Instruments) equipped with 20× objective lens, which can take high-definition phase-contrast and green fluorescence images in real time. Images were analyzed using IncuCyte Software. For immunofluorescent staining, 10,000–20,000 MCF10Ca1h cells were plated onto Borosilicate Chambered Coverglass (Lab-Tek) for 1–5 days in regular growth medium and then fixed and immunostained for cytokeratin markers as detailed in Supplemental Experimental Procedures.

### Asymmetric Division

MCF10Ca1h transduced with Sore6-GFP were cultured in 1 μM BrdU (Sigma) containing cell culture medium for 2 weeks to ensure all cells were labeled with BrdU. Cells were then sorted for GFP-positive and GFP-negative cells. Sorted cells were cultured for two cell divisions in the absence of BrdU (the chase) and then collected by mitotic shake-off for analysis of mitotic pairs with asymmetrically distributed BrdU label as described previously (Pine et al., 2010), with more details in Supplemental Experimental Procedures.

### Tumorsphere Formation and Cell Invasion Assays

To assess tumorsphere-forming ability, single-cell suspensions of tumor cells were plated in ultra-low-attachment 24-well plates (Corning) at 2,500 cells/well in regular growth medium. After 5–7 days, wells were examined under an inverted microscope at ×40 magnification, and the number of spheres of >100 μm in diameter were counted for a total of 15–20 independent fields per well and three replicate wells per condition. Confocal images of representative tumorspheres were acquired using a Zeiss 710 confocal microscope (Carl Zeiss). Cell invasion assays were carried out using the Growth Factor Reduced BD Matrigel Invasion Chamber (8 μm, BD Biosciences). A total of 5,000 cells were plated in each chamber in normal growth medium for 3 days. The cells on the upper side of the membrane were removed while the cells on the lower side were methanol-fixed and stained with 0.05% crystal violet. The invaded cells were counted under an inverted microscope at ×20 magnification, for a total of 20–25 independent fields per well and three replicate wells per condition.

### Confocal Imaging

Confocal imaging of freshly excised tumors and lungs was done using a Zeiss 780 Confocal microscope setup with 405, 488, and 561nm lasers. Confocal images were sequentially acquired with Zeiss ZEN software on a Zeiss LSM Confocal system (Carl Zeiss). For the deeper optical sections (250 μm) in excised tumors, a Zeiss 710 upright confocal microscope was used.

### Statistical Analysis

Statistical analyses were done using the statistical tools in GraphPad Prism 5.0 (GraphPad Software). Specific tests used are indicated in the text. p < 0.05 was considered significant.

Additional methodological details can be found in Supplemental Experimental Procedures.

## Figures and Tables

**Table 1:** In Vivo Limiting Dilution Assay for MCF10Ca1h Cells

Cell Population	No. of Cells Implanted/Site				1/CSC Frequency	95% CI
	5,000	2,500	500	100		
	Tumor Incidence					
Sham sort	5/6	4/6	1/6	1/6	2,343	4,564–1,203
SORE6^−^	2/6	1/6	0/6	0/6	14,308	44,186–4,633
SORE6^+^	6/6	5/6	4/6	3/6	722	1,497–438

The indicated number of cells was implanted orthotopically into nude mice, and tumor incidence was assessed after 3 months. CSC frequencies were calculated using ELDA software. CI, confidence interval.

**Table 2:** Enrichment for Tumor-Initiating Cells in SORE6^+^ Fractions from Three Human Breast Cancer Cell Lines

Cell Line	Breast Cancer Subtype	CSC Frequency SORE6^−^	CSC Frequency SORE6^+^	Enrichment Factor	p Value
MCF7-EP	ER+	1/9,097	>1/430	>21.1	1.2 × 10^−09^
MCF10Ca1h	ER+	1/14,308	1/722	19.8	6.9 × 10^−08^
MDA-MB-231	TNBC	1/549	1/58	9.5	4.5 × 10^−08^

Tumor cells at different dilutions were implanted orthotopically into nude mice, and tumor incidence was assessed after 1–3 months, depending on the model. CSC frequencies were calculated using ELDA software. p value is for chi-square test. ER, estrogen receptor; TNBC, triple-negative breast cancer.

## Supplemental Information

Document S1. Supplemental Experimental Procedures and Figures S1–S3

Movie S1. Time-Lapse Videomicroscopy of SORE6^+^ MCF10Ca1h Cells Growing in Culture, Related to Figure 2 A FACS-sorted, SORE6^+^ enriched MCF10Ca1h cell population was seeded at low density and imaged every 2 hours over ∼5 days. For viewing guidance, see freeze-frame images at different time points in Figure 2D and accompanying description in the figure legend

Movie S2. Time-Lapse Videomicroscopy of the Effect of Paclitaxel Treatment of Unsorted MCF10CA1h Cultures Transduced with SORE6-GFP, Related to Figure 4 Cells were treated with 50 nM doxorubicin at t = 0 hr and imaged every 3 hours over 39 hours. SORE6- cells begin to die at t = ∼9 hr.

Movie S3. Time-Lapse Videomicroscopy of Untreated MCF10CA1h Cultures over the Same Time Period, Related to Figure 4 As for Movie S2, but cells were treated with vehicle only.

Document S2. Article plus Supplemental Information
