# Supplementary material for: A Flexible Reporter System for Direct Observation and Isolation of Cancer Stem Cells
Source: Stem Cell Reports. 2014 Dec 11;4(1):155–69. doi: 10.1016/j.stemcr.2014.11.002 (PMC4297872; doi:10.1016/j.stemcr.2014.11.002)
Supplement: Document S1. Supplemental Experimental Procedures and Figures S1–S3 [file mmc1.pdf]

Stem Cell Reports, Volume *4*

Supplemental Information

A Flexible Reporter System for Direct Observation and Isolation of Cancer Stem Cells

Binwu Tang, Asaf Raviv, Dominic Esposito, Kathleen C. Flanders, Catherine Daniel, Bao Tram Nghiem, Susan Garfield, Langston Lim, Poonam Mannan, Ana I. Robles, William I. Smith, Jr., Joshua Zimmerberg, Rea Ravin, and Lalage M. Wakefield

SUPPLEMENTAL DATA

Supplemental Figure S1. SORE6 Reporter Activity Depends on Endogenous SOX2 and OCT4 Expression Which Increases with Increasing Malignancy, Related to Figure 1.
A. *SOX2* and *OCT4* were knocked down in MCF7 cells by reverse transfection with ON-TARGETplus siRNAs. Knockdown efficiency was assessed by RTQ-PCR. Results are mean +/- SD for 3 independently transfected cultures. *, p<0.05, t-test. **B.** FACS analysis showing reduction in SORE6+ fraction following partial knockdown of *SOX2* and *OCT4*. Results are mean +/- SD for 3 independently transfected cultures. *, p<0.05; ** p<0.01, one-way ANOVA with Dunnett's multiple comparison test. **C.** Immunofluorescent staining of MCF10Ca1h cells for OCT4, showing the presence of OCT4 (pink) in all cells expressing the SORE6 reporter (green). Scale bar, 20μm. **D.** *SOX2* and *OCT4* mRNA expression in different breast cancer cell lines assessed by RTQ-PCR. Results are normalized to *PPIA* for each cell line and presented as mean +/- SD for 3 independent experiments. The percentage of SORE6+ cells in the population is shown for comparison.

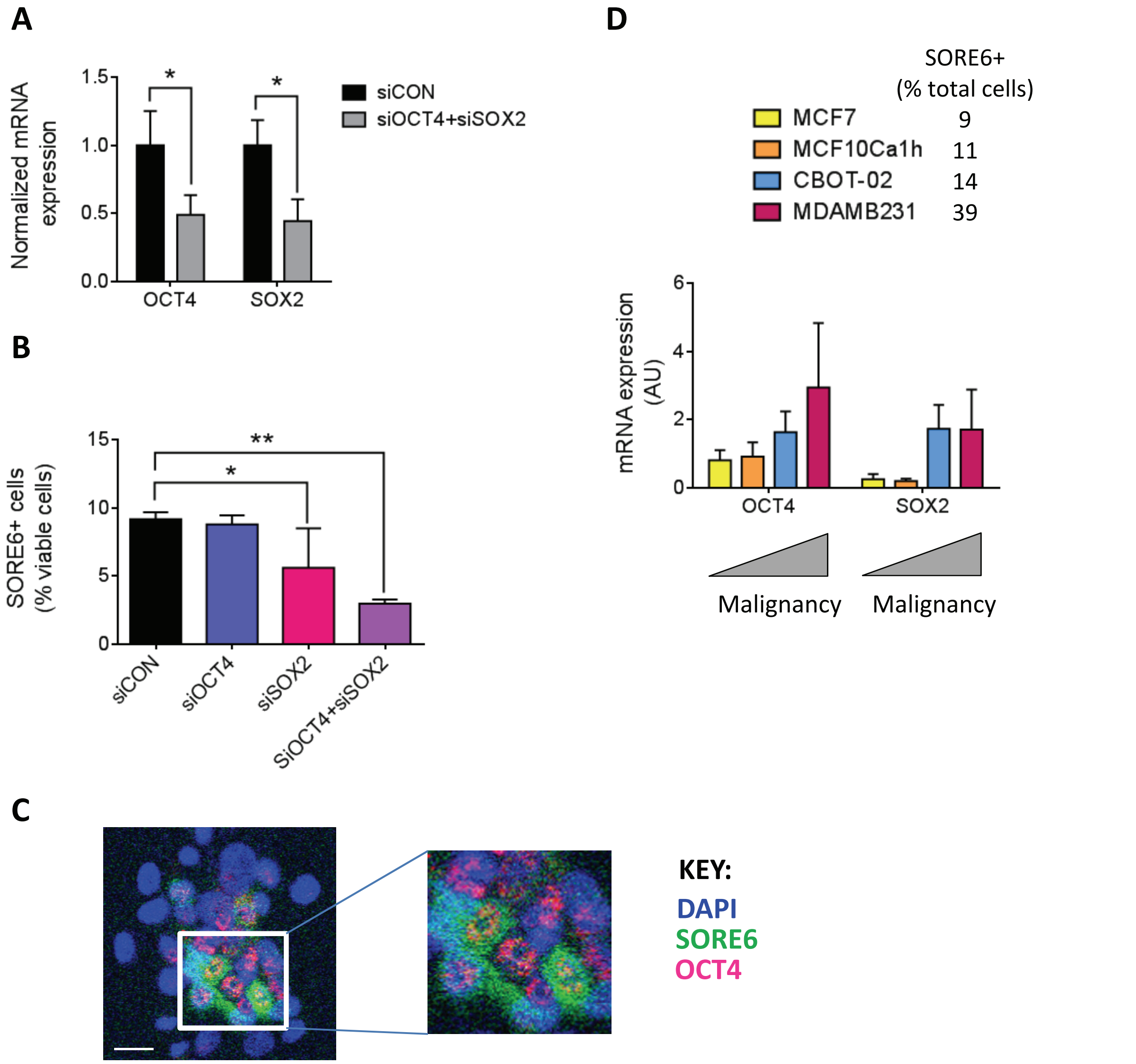

Supplemental Figure S2. The Ornithine Decarboxylase Degron Sequence that Destabilizes the Fluorescent Protein Increases the Selectivity of the SORE6 Reporter for the CSC Population, Related to Figure 2. A. MCF7 and CBOT2 cells stably transduced with the SORE6 reporter were treated with the proteasome inhibitor MG132 (1μM) for 24h and the relative proportion of SORE6+ cells was assessed by FACS analysis. Results are the mean +/- SD for 3 independent cultures of each cell line analyzed in one experiment. **B.** MCF7 cells stably transduced with the SORE6 reporter were treated with proteasome inhibitor as above and the effect on tumorsphere formation efficiency was assessed. Results are the mean +/- SD for 3 independent cultures analyzed in one experiment. **C.** Western blot showing efficacy of MG132 treatment in inhibiting the proteasome as indicated by the increase in ubiquitinated protein in the cultures.

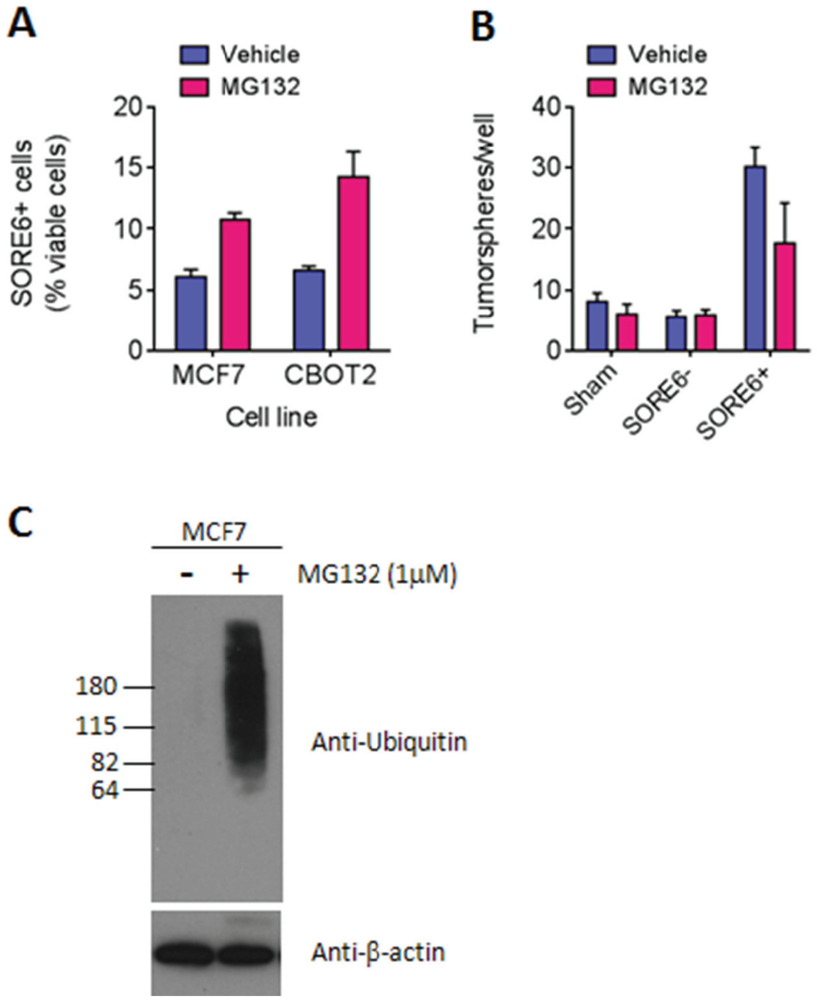

Supplemental Figure S3. Relationship between SORE6+ Population and Cell Populations Identified using other Breast Cancer Stem Cell Markers, Related to Figure 2. A. Unsorted cell cultures and FACS-sorted SORE6+ cell populations were analyzed for CD24 and CD44 cell surface marker expression by FACS to address whether the CD44+CD24- population was enriched in the SORE6+ fraction. The CD44+CD24-/lo fraction (red) has been shown to enrich for CSCs in some breast cancer models. The distribution of cells between the different CD44CD24 marker combinations is represented in the pie-charts. The data in each pie chart represent the mean results for 3 independent cultures for each cell type analyzed. **B.** MCF10Ca1h cells stably transduced with the SORE6 reporter (green) were immunostained for ALDH1A1 (red). Representative image in which arrows indicate double positive cells and arrowheads indicate single positive ALDH1+ cells. Although positive cells were rare in these cultures, SORE6+ cells were nearly all ALDH1+, but ALDH1+ cells were not always SORE6+.

A

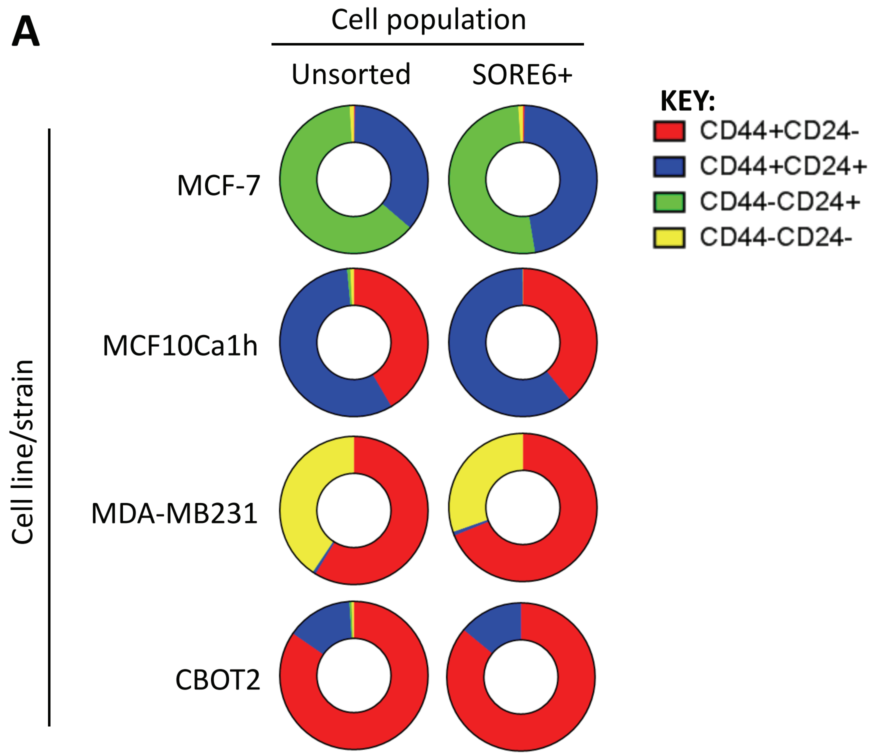

B

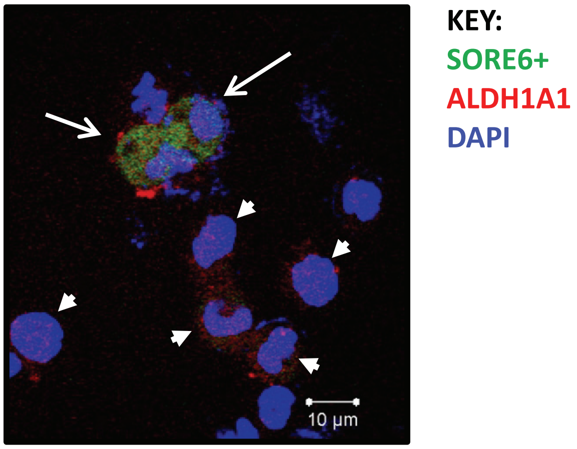

SUPPLEMENTAL EXPERIMENTAL PROCEDURES.

Breast cancer primary cultures. Anonymized primary breast cancer specimens were obtained from Suburban Hospital, Bethesda MD, under an NIH Office of Human Subjects Research exemptions (OHSR # 11736) that allows for the use of tumor material as discarded medical waste. CBOT1 was a poorly differentiated ductal carcinoma, grade 3, *pT2, pN2, Mx,* Stage IIIA, ER+, PR+, HER2-. CBOT2 was a moderately differentiated ductal carcinoma, grade 2, *pT1a, N0, Mx,* Stage 1A, ER+, PR unknown, HER2+. CBOT3 was a moderately differentiated multifocal ductal carcinoma, grade 2, *pT1c, N0, Mx*, Stage 1A, ER+, PR+, HER2-. Specimens were cut into small pieces a few mm in diameter. For CBOT1, each tissue slice was placed on top of a single 1.5X0.7-cm piece of sterile Gelfoam (Pfizer-Pharmacia & Upjohn Co) that had been preincubated for 1 hour in 6-well tissue culture plates with 2 ml culture medium (DMEM/F12 with 10% FBS, 1% penicillin/streptomycin). The tumor sections were placed in a humidified 5% CO_2 incubator at 37°C for 2 weeks. Fresh culture medium was replaced every 3-4 days. During the incubation, tumor cells migrated through the Gelfoam to the bottom of the 6 well plates and grew there. The Gelfoam was removed from the plate, and tumor cells were maintained in culture for passaging and lentiviral infection. For CBOT2 and CBOT3, tumors were minced and digested with DMEM/10% FBS containing 1 mg/ml collagenase I (Sigma), 1 mg/ml collagenase D (Sigma) and 1mg/ml DNase I for 1 hour at 37°C, and then cells were filtered through a 40μm cell strainer, harvested by centrifugation, washed in PBS and suspended and cultured in DMEM, 10% FBS. With either tumor cell preparation procedure, fibroblasts were removed by light trypsinization, and the composition of the cultures that were used for experiments was determined to be >95% epithelial by immunostaining for epithelial cytokeratins with an anti-human pan-cytokeratin antibody (Clone AE1/AE3; #M3515, DAKO, Carpintaria, CA)

Asymmetric Division.
MCF10Ca1h transduced with Sore6-GFP were cultured in 1μM BrdU (Sigma) containing cell culture medium for 2 weeks to ensure all cells were labeled with BrdU. Cells were then sorted for GFP positive and negative cells. Sorted cells were cultured for two cell divisions in the absence of BrdU (the chase), and then collected by mitotic shake-off for analysis of mitotic pairs with asymmetrically distributed BrdU label as described (Pine *et al.*, 2010), To reveal BrdU-labeled DNA, cells were fixed by ice-cold 70% ethanol for overnight in -20°C and incubated in 2N HCL containing 0.5% Triton-X-100 for 1 h. Cells were then washed with PBS containing 0.1% BSA and 0.5% Triton-X-100 and then in PBS with only 0.1% BSA. Cells were then incubated with a 1:5 dilution of anti-BrdU-FITC antibody (BD Biosciences) for 1 h at room temperature. After two washes with PBS, cells were cytospun onto glass microscope slides with 10,000 cells per slide, and then mounted using Vectashield containing DAPI (Vector Laboratories). Confocal images were acquired with Zeiss ZEN 2009 software on a Zeiss LSM 710 NLO Confocal system (Carl Zeiss Inc, Thornwood, NY) with a Zeiss Observer Z1 inverted microscope and diode laser tuned to 405 nm, a 25 mW Argon visible laser tuned to 488 nm. Cells were scored if both sets of chromosomes were clearly separated and had parallel and condensed chromatin. The cell scored as asymmetric partitioning of BrdU was the cell which one set of chromosomes was strongly positive and the other set was completely negative, as determined by careful visualization under 40X magnification with Z-stack setting. 30-50 mitotic

daughter pairs were counted for each experimental condition. 3 independent experiments were performed and results are expressed as the mean +/- SEM for the 3 experiments.

Immunofluorescence.
Cells were fixed with 1% PFA for 10 minutes at room temperature and 2 minutes in ice cold acetone. Cells then were incubated in blocking buffer with 5%BSA and 0.5% Tween-20 in PBS for 1 hour at room temperature. Primary antibodies were incubated in blocking buffer overnight at 4^oC. Cells were washed with PBS following incubation with secondary antibodies for 45 minutes in room temperature. The primary antibodies used were: anti-cytokeratin 5 (rabbit,1:1000, PRB-155P, Covance), anti-cytokeratin 8 (rat, 1:200, TROMA-1, Hybridoma Bank, University of Iowa), anti-cytokeratin 14 (rabbit, 1:5000, PRB-160P, Covance), anti-OCT4 (mouse, 1:500, SC5279, Santa Cruz), anti-ALDH1A1 (mouse, 1:500, #611194, BD Biosciences). The following secondary antibodies were used: anti-rat, anti-mouse and anti-rabbit conjugated to AlexaFluor 594 (Molecular Probes). Nuclei were stained with DAPI mounting medium (Vector). Images were acquired using a Zeiss 510 confocal microscope.

SOX2 and OCT4 overexpression and knockdown.
For SOX2 and OCT4 overexpression, lentiviruses from the Stemgent® Lentivirus Set (hOSLN: Cat # ST000005, Sigma-Aldrich) were used. Cells were exposed to virus for 24 hours and used without selection. For SOX2 and OCT4 knockdown, cells were reverse transfected with ON-TARGETplus siRNAs from Dharmacon (OCT4, L-019591-00-0005; SOX2, L-011778-00-0005) and analyzed after 72 hours.

RTQ-PCR. Total RNA was extracted RNeasy Mini Kit (Qiagen). Real-time PCR was done using the SuperScript III First Strand Synthesis System (Invitrogen) to generate cDNAs from RNA samples, followed by PCR in a fluorescent temperature cycler (Bio-Rad) using Brilliant SYBR Green QPCR Master Mix (Agilent Technologies). *PPIA* was used as a reference transcript for normalization. The *OCT4* primer pair specifically detects *OCT4* and not *OCT4* pseudogenes (Atlasi *et al.*, 2008).

Primers used were as follows:
SOX2-F 5'-TAAATACCGGCCCCGGCGGA-3'
SOX2-R 5'-TGCCGTTGCTCCAGCCGTTC-3'
OCT4-F 5'-CTTCTCGCCCCCTCCAGGT-3'
OCT4-R 5'-AAATAGAACCCCCAGGGTGAGC-3'
NANOG-F 5'-ACATGCAACCTGAAGACGTGT-3'
NANOG-R 5'- CATGGAAACCAGAACACGTGG-3'
PPIA-F 5'-GTCAACCCCACCGTGTTCTT-3'
PPIA-R 5'-CTGCTGTCTTTGGGACCTTGT-3' .

Detailed construction of Gateway-based cancer stem cell reporter pro-lentiviral plasmids.

Summary. Lentiviral reporter constructs were made by Gateway Multisite recombinational cloning of the SORE6 minigene, minimal CMV promoter, and destablized fluorescent proteins. Each element was separately cloned and sequence verified prior to Gateway assembly into pDest-663, a lentiviral Destination vector based on the pFUGW lentiviral backbone with

puromycin selection. In addition to dsCopGFP, a new destabilized form of monomeric Cherry fluorescent protein (dsmCherry) was constructed by addition of the PEST destabilization sequence (degron) from the ornithine decarboxylase gene to the C-terminus of mCherry. Constructs were also generated in which the full CMV promoter was used to drive constitutive expression of fluorescent proteins for labeling all cells in the culture.

SORE6 minigene. The sequence of the SORE6 minigene containing 6 tandem repeats of the composite SOX2/OCT4 response element is given below, with the SOX2/OCT4 response element in bold.

5’atctatcgatcagctac**ttttgcattacaatg**gccttggtgcagctac**ttttgcattacaatg**gccttggtgcagctac**ttttgcattacaa**
tggccttggtggaattccagctac**ttttgcattacaatggccttg**gtgcagctac**ttttgcattacaatg**gccttggtgcagctac**ttttgca**
ttacaatggccttggtgactagttcta-3’.

Oligonucleotides and Plasmids. pDonr253 is a Gateway Donor vector modified from pDonr201 (Life Technologies). pDonr253 replaces the kanamycin resistance gene with a gene encoding spectinomycin resistance, and contains several sequencing primer sites to aid in sequence verification of Entry clones. pDonr215 is a modified version of pDonr201 which contains altered Gateway recombination sites (attP4 and attP5). pDonr235 is a modified version of pDonr253 with attP5r and attP1r sites. pDonr233 is a modified version of pDonr253 with attP4 and attP1r sites. The following oligonucleotides (Eurofins MWG Operon, Inc) were used in this study:

7560: 5’-GGGGACAACTTTGTATAGAAAAGTTGGCCAAATTACAAAATTCAAAATTTTATCGATC
7561: 5’-GGGGACAACTTTGTATACAAAAGTTGGCCCGTACACGCCTAACTAGTCAC
7562: 5’-GGGGGCCAACTTTTGTATACAAAGTGGTAGGCGTGTACGGTGGGAGGCC
7563: 5’-GGGGCCAACTTTTTTGTACAAAGTTGTGCTGGATCGGTCCCGGTGTCTTC
8067: 5’-GGGGACAACTTTGTACAAGAAAGTTGATTACACATTGATCCTAGCAGAAGCACAGGC
8105: 5’-GGGGACAACTTTGTACAAAAAAGTTGGCACCATGCCCGCCATGAAGATCGAGTGCCG
8580: 5’-
GACATGGGCAGCGTGCCATCATCCTGCGCCGCCACCGCCGGCGGGAAGCCATGGCTAAGCTTGTACAG
CTCGTCCATGCC
8581: 5’-
CCTAGCAGAAGCACAGGCTGCAGGGTGACGGTCCATCCCGCTCTCCTGGGCACAAGACATGGGCAGC
GTGCCATCATCC
8582: 5’-GGGGACAACTTTGTACAAGAAAGTTGATTACACATTGATCCTAGCAGAAGCACAGGCTGC
8583: 5’-GGGGACAACTTTGTACAAAAAAGTTGGCACCATGGTGAGCAAGGGCGAGGAG
11151: 5’-GGGGACAACTTTGTATAGAAAAGTTGGCTAGGCGTGTACGGTGGGAGGCC

Generation of Entry clones. Entry clones were constructed by PCR amplification of DNA sequences flanked by Gateway Multisite recombination sites (Life Technologies, Carlsbad, CA). PCR was carried out with 200 nM of each oligo listed in the table below using Phusion polymerase (New England Biolabs) under standard conditions and an extension time of 30 seconds for 20 cycles. PCR products were cleaned using the QiaQuick PCR purification kit (Qiagen, Valencia, CA). The final PCR products were recombined into the approriate Gateway Donor vector using the Gateway BP recombination reaction using the manufacturer’s protocols. The subsequent Entry clones were sequence verified throughout the entire cloned region.

For clone 8522-E16, the PEST destabilizing sequence was attached to the C-terminus of mCherry using an adapter PCR approach. After 5 cycles of PCR with the 5’ and 3’ oligos,

adapter oligo 8581 was added and amplification was continued for another 5 cycles. Following this, the 8582 adapter oligo was added and amplification was continued for an additional 15 cycles.

Entry clone	Sequence	GW sites	5' oligo	3' oligo	adapters	template	Donor
8138-E02	SORE6 minigene	attB4-attB5	7560	7561		pTRH1-SORE6	pDonr215
8138-E03	mCMV promoter	attB5r-attB1r	7562	7563		pCMV-Sport6	pDonr235
8138-E04	dsCopGFP	attB1-attB2	8105	8067		pTRH1-SORE6	pDonr253
8522-E16	dsmCherry	attB1-attB2	8583	8580	8581/8582	pRSET-mCherry	pDonr253
C413-E34	mCMV promoter	attB4-attB1r	11151	7563		pCMV-Sport6	pDonr233

Subcloning for lentiviral reporter constructs. Gateway Multisite LR recombination was used to construct the final lentiviral reporter constructs from the Entry clones using the manufacturer's protocols (Life Technologies, Carlsbad, CA). The Gateway Destination vector used is pDest-663, a lentiviral vector containing a Gateway attR4-attR2 cassette based on a modified version of the pFUGW lentiviral vector which contains the enhanced polypurine tract (PPT) and woodchuck regulatory element (WRE) to provide higher titer virus. In addition, it contains an antibiotic resistance gene for puromycin resistance. Final expression clones were verified by restriction analysis and maxiprep DNA for lentivirus production was prepared using the GenElute XP purification kits (Sigma, St. Louis, MO). Final clones contained combinations of Entry clones as follows:

Expression clone	Description of inserts	Short clone name	Entry 1	Entry 2	Entry 3
8522-M01-663	SORE6-minCMV-dsCopGFP	SORE6-GFP	8138-E02	8138-E03	8138-E04
8522-M31-663	SORE6-minCMV-dsmCherry	SORE6-mCherry	8138-E02	8138-E03	8522-E16
10279-M28-663	minCMV-dsCopGFP	minCMV-GFP	C413-E34	8138-E04	
10279-M30-663	minCMV-dsmCherry	minCMV-mCherry	C413-E34	8522-E16	
8522-M10-664	CMV32p-eGFP	CMV-eGFP	C413-E08	C122-E11	
8522-M09-685	CMV32p-mKate	CMV-mKate	C413-E08	C122-E14	

SUPPLEMENTAL REFERENCES

Atlasi Y, Mowla SJ, Ziaee SA, Gokhale PJ, and Andrews PW (2008) OCT4 spliced variants are differentially expressed in human pluripotent and nonpluripotent cells. *Stem Cells,* **26,** 3068-3074.

Pine SR, Ryan BM, Varticovski L, Robles AI, and Harris CC (2010) Microenvironmental modulation of asymmetric cell division in human lung cancer cells. *Proc Natl Acad Sci U S A,* **107,** 2195-2200
